# Supplementary material for: Post-HPV-Vaccination Mast Cell Activation Syndrome: Possible Vaccine-Triggered Escalation of Undiagnosed Pre-Existing Mast Cell Disease?
Source: Vaccines (Basel). 2022 Jan 16;10(1):127. doi: 10.3390/vaccines10010127 (PMC8779641; doi:10.3390/vaccines10010127)
Supplement: Supplementary file 1 [file vaccines-10-00127-s001.zip › vaccines-1505408-sup-proofed/vaccines-1505408-sup-proofed.pdf]

# Post-HPV-Vaccination Mast Cell Activation Syndrome: Possible Vaccine-Triggered Escalation of Undiagnosed Pre-Existing Mast Cell Disease?

Online Supplement: Detailed Case Histories

Lawrence B. Afrin, M.D.,[1] Tania T. Dempsey, M.D.,[1]

Leonard B. Weinstock, M.D.[2]

[1]AIM Center for Personalized Medicine, Purchase, New York, United States

[2]Department of Medicine, Washington University School of Medicine,

St. Louis, Missouri, United States

January 15, 2022

Case 1

This female, who was 18 at the time of her initial presentation for evaluation for MCAS, was one of a premature twin delivery, and though there was mild developmental delay in her first several months, by 1-1.5 years, she had "caught up." She appeared to be fully healthy for the next several years, but starting around age 8, she began suffering frequent spontaneous nosebleeds. Annual check-ups with her pediatrician never identified a cause for these events. Around age 10 she started suffering bilateral knee pain. She soon was diagnosed with Osgood-Shlatter disease. She was casted for about two months, but this did not help. Menarche came at 12 and was unremarkable. Her early adolescence was otherwise unremarkable except for acne which emerged around the same time (age 12), but then, around age 16 in 2016, about two months after she began taking chronic erythromycin to treat acne (note the treatment did not seem to help the acne), her feet became erythematous and blistered and started feeling like they were "burning from the inside out." A biopsy of the erythema by a dermatologist found only non-specifically inflamed tissue. At 17, about six weeks after her first Gardasil vaccination (which she had appeared to acutely tolerate without difficulty), one night, "out of the blue," she started suffering palpitations and pulsatile tinnitus. Her mother, a nurse, checked her pulse the next morning and found it to be about 160; the patient also was flushed at the time. About 30 seconds into that pulse count, there was a sudden pause in the tachycardia, at which point the patient suffered an acute full syncope. She was taken to a nearby emergency department (ED). While there, she continued suffering waxing/waning flushing. While on a heart monitor, she was manifesting multiple runs of non-sustained ventricular tachycardia, which were witnessed only by the patient's mother. She also was hypertensive, about 140/90. Orthostatic hypotension testing was positive. She was admitted to the hospital. An echocardiogram was unremarkable. A consulting

cardiologist suspected postural orthostatic tachycardia syndrome (POTS), which did not entirely make sense to the patient's mother since there clearly was some non-orthostatic component to the cardiac issues. She was referred to a nephrologist (because of the hypertension) to rule out a pheochromocytoma, but testing (principally an ultrasound and blood and urine testing) was all negative except for a slight increase in norepinephrine. Many more symptoms came on in the days and weeks beyond that point, including chest pain and palpitations, numbness and tingling in her distal extremities, tremors, difficulty focusing her vision, cognitive dysfunction (especially memory, also occasionally almost catatonic, according to her mother), insomnia, anorexia, diffusely migratory joint and bone pain, and severe fatigue. There were times when she could barely walk because of all of these symptoms. About five months after the initial syncope, she finally was able to consult with a neurologist, and she was immediately suspected of having hypermobile Ehlers Danlos Syndrome (hEDS). She had a 9/9 Beighton score and was diagnosed with hEDS. Her neurologist also shortly suspected she also had a mast cell activation syndrome (MCAS) and referred her to an immunologist to investigate this suspicion. Meanwhile, she was empirically started by the neurologist on guanfacine. On the second day of this trial, she developed acute numbness of the bilateral calves and toes. The guanfacine was immediately stopped by the neurologist. She also was then started on Zyrtec and Zantac and oral cromolyn. She also underwent a tilt table test which showed "possible" hyperadrenergic postural orthostatic tachycardia syndrome (hyperPOTS), with her blood pressure peaking around 190/103, causing significant acute visual disturbance. She then consulted with an immunologist for evaluation of suspected MCAS, who felt her presentation was consistent with MCAS but was unsure how to proceed with diagnostic testing. The patient did not think that cromolyn was helping, so cromolyn was stopped and ketotifen was added. The immunologist then performed skin patch

food allergy testing on her, finding an extensive array of positives, and yet she knew she did not react to any of the foods which showed positive reactivity on the testing. Also, she was beginning to find an increasing array of modest stimuli which were provoking flares of flushing and other symptoms. She started developing sensitivities to various odors, and in 1/18 she was exposed to a car air freshener and acutely developed hives, headache, flushing, dyspnea, and throat numbness. She returned to the immunologist and was prescribed montelukast 10 mg once daily plus an epinephrine autoinjector as needed for anaphylaxis. She improved only slightly once montelukast was started. Within a few months, her array of symptoms had worsened to the point where she could not walk more than a hundred feet without having to sit and rest. She was continuing to suffer chest pain, tachycardia, and palpitations with even the slightest exertion. She soon consulted an electrophysiologist. A Holter monitor led to a diagnosis of inappropriate sinus tachycardia (IST). She was started on diltiazem and midodrine, which helped her fatigue somewhat (she was now able to walk a bit longer before requiring rest) but not the other symptoms. Her local physicians at this point, seeing little improvement, wanted to start weaning her off some of her medications, but her mother was concerned this would leave the patient even more debilitated. At this point (especially with the patient having just gotten bone densitometry which suggested osteopenia despite there being no apparent cause), her mother began wondering whether other diagnostic and therapeutic efforts might be needed with regard to the patient's clinically diagnosed MCAS, ultimately leading to referral to author LBA. Her chief complaints at the time of initial evaluation were episodes of chest pain, cognitive dysfunction, joint pain, and epistaxis. Her mother further noted that when the patient suspended her antihistamines to undergo allergy testing, she developed another episode of epistaxis that very night.

On a full review of systems at the initial evaluation by LBA, although she denied any issues with diaphoresis, irritation of the eyes/nose/mouth/throat, easy bleeding (other than epistaxis), sinonasal congestion, coryza, post-nasal drip, dysphagia, wheezing, gastroesophageal reflux, vomiting, diarrhea, constipation, abdominal discomfort/pain, bloating, genitourinary problems, edema, dental/gingival problems, onychodystrophy, she endorses a wide range of other, chronic/recurrent, episodic/intermittent and/or waxing/waning issues including subjective (rarely objective) fevers, flushing, feeling cold some of the time, fatigue (often to the point of exhaustion), malaise, headaches, diffusely migratory aching/pain, diffusely migratory pruritus, unprovoked fluctuations in weight, poor appetite, acute brief inability to focus vision, tinnitus, epistaxis, easy bruising, dyspnea with flares, palpitations, non-anginal chest discomfort/pain, nausea, diffusely migratory weakness, diffusely migratory tingling/numbness (typically about the distal extremities), diffusely migratory bilateral cervical adenopathy and adenitis, orthostatic and non-orthostatic presyncope, syncope twice (the unprovoked incident detailed above and once more at the time of a blood draw), cognitive dysfunction (particularly memory, concentration, and word-finding), possible hair loss, diffusely migratory rashes (typically patchy macular erythema, and particularly about the toes with associated burning), hives, insomnia, non-restorative sleep, suspected (but not yet formally diagnosed) anxiety and depression, modest joint hypermobility (she spontaneously demonstrated a Beighton score of only 4/9 at the thumbs and little fingers), unusually vigorous reactions to insect bites, and poor healing in general.

Family history was notable for a mother with sarcoidosis and suspicions of MCAS, a sister who had Stage II nodular sclerosing Hodgkin lymphoma at 12 and quickly achieved a remission which had persisted ever since but who was also now suspected of also having MCAS, a

maternal grandfather who had a brain aneurysm, a maternal grandmother who had bladder cancer, paternal grandparents who were healthy, a father who was healthy, and two other siblings who both had hEDS. The patient denies any history of smoking, significant alcohol use, or illegal substance use.

At the time of the initial evaluation by LBA, the patient listed her current medications as diltiazem 30 mg thrice daily, ranitidine 150 mg twice daily, cetirizine 10 mg twice daily, midodrine 5 mg twice daily, hydroxyzine 25 mg as needed, compounded oral ketotifen 1 mg twice daily, and montelukast 10 mg once daily. She denied any known drug allergies.

Physical examination at the initial evaluation by LBA found a modestly acneiform and vaguely tired-appearing but otherwise outwardly healthy appearing, trim young woman in no acute distress, pleasant, cooperative, fully alert and oriented, easily independently ambulatory, presenting with her appropriately concerned and supportive mother. Vital signs were notable for blood pressure 128/93, pulse 95, weight 105 pounds. Key findings were her mildly tired/acneiform but otherwise comfortable general appearance, head/ears/eyes/nose/throat (HEENT) benign, no plethora/pallor/diaphoresis/jaundice/bleeding/bruising, a modest extent of migratory waxing/waning small-patchy macular erythematous rash scattered about the anterior neck, supple, no jugular venous distention (JVD) or thyromegaly or carotid/abdominal/renal bruits (but she did have mild bilateral femoral bruits), no palpable adenopathy or tenderness at any of the usual node-bearing sites, clear lungs, regular heart with no adventitious sounds, abdomen completely benign, zero peripheral edema, and the neurologic examination, too, was unremarkable. No costovertebral angle or spinal column tenderness was evoked on palpation

and percussion about these areas. On a light scratch test on the upper back, moderately bright dermatographism (erythroderma only, no hives) quickly emerged but was beginning to resolve when last checked 10 minutes later.

Laboratory testing for mast cell activation was positive for a 24-hour urinary prostaglandin $D_2$ level elevated at 777 ng (normal 100-280) and a plasma heparin level elevated at 0.07 anti-Factor Xa units/ml (upper normal 0.02 [1]).

Trials of various non-sedating $H_1$ blockers and various $H_2$ blockers found loratadine and ranitidine served her better than the others, having gained her a broad range of symptomatic improvements at follow-up three months after initial evaluation. She was subsequently lost to follow-up.

Case 2

This female, who was 19 at the time of her initial presentation for evaluation for MCAS, was healthy in her first year of life, but at age 1, shortly after starting amoxicillin for her first earache, she suffered a diffuse outbreak of hives. By the time she started kindergarten at 4, she was already showing unusual anxiety. She routinely complained even at that age of difficulty swallowing and breathing. Her maternal grandfather died when the patient was 5, and soon after this she appeared to develop "an irrational fear of dying," and she soon began suffering panic attacks. From about age 6 to 10, she routinely suffered severe idiopathic anxiety attacks. Around age 11, a few months after she started attending a new school (after having left the prior

school due to having suffered a bullying incident), she suffered four straight weeks of vomiting. Menarche came a few weeks later and was unremarkable. At 13 she suffered heat stroke while playing field hockey. By age 14 she was suffering substantial dysmenorrhea. At 15 she got her first Gardasil vaccination, and two months later she began suffering severe fatigue and developed a new pruritic oral sensitivity to a variety of foods. She also developed persistent non-bloody diarrhea. Multiple evaluations by her pediatrician, including blood and stool testing, were all unrevealing except for reportedly showing evidence of prior Epstein-Barr virus infection. She was told by multiple providers that her problems were all psychosomatic. She continued on schedule with the second and third Gardasil vaccinations. At 16, she was referred to a pediatric gastroenterologist, who performed upper endoscopy and diagnosed eosinophilic esophagitis (EoE). Other than PPIs (which did not help any of her symptoms) and budesonide, no specific treatment was recommended, and she found that budesonide (and, later, prednisone, too) caused extreme exhaustion, irritability, and hunger, and overall she "felt like death" while on budesonide or prednisone. She then consulted a naturopath who recommended she eliminate "the top 8 allergens" from her regimen, a change which helped only "a little bit," but at the same time she started developing other issues such as brain fog, severe joint and muscle pains, temperature sensitivities, hot flashes, freezing episodes, and marked worsening of anxiety. She also started suffering presyncopal and syncopal episodes. She also had impacted wisdom teeth extracted around this time and about a month later suffered a bout of lockjaw followed by infection at one of the extraction sites. She was given codeine for this but found it ineffective, and actually it only seemed to cause marked weakness and memory loss. She then was evaluated at a center for eosinophilic disorders. The diagnosis of EoE was confirmed, and she was told she might actually have eosinophilic gastroenteritis, so bidirectional endoscopy was pursued, which

found no eosinophils in either the upper or lower gastrointestinal tract, an improvement attributed to the dietary changes she had already implemented. Her consultant was not convinced at that point that she had EoE, so he recommended she drink milk for three months and then undergo repeat endoscopy, but she knew this would make her sick, so she demurred. She then underwent allergy testing and was told by those evaluators that they had never before seen anybody so allergic. She pursued a severe elimination diet, which resolved her diarrhea but did not help any of her other symptoms. At 17 she relocated to see if a new environment might be helpful, which helped somewhat for some of her symptoms, but it was stressful living with an alcoholic relative. She also was evaluated by a gynecologist shortly before moving and was told she might have polycystic ovarian syndrome (PCOS), for which a daily oral contraceptive was started. The contraceptive quickly resolved the dysmenorrhea but did not seem to help any of her other symptoms. At 18 she went to college. None of her symptoms improved. Joint pains began worsening. She started suffering severe headaches. She found she was markedly intolerant of gluten and soy, exposure to which would acutely exacerbate many of her symptoms. She decided to switch schools after the first year due to bullying there, too. A relative first suggested to her that mast cell activation disorder might be at the root of the patient's symptoms, soon leading to evaluation by author LBA. Her chief complaints at the time of the initial evaluation were fatigue, joint and muscle pains, food sensitivities, and brain fog. Past medical history obtained at that time was additionally notable for hair loss, nausea, vomiting, cognitive dysfunction, abdominal pain, infantile eczema, allergic rhinitis, drug allergy, gastroesophageal reflux, epistaxis, and chest pain. On a full review of systems at the initial evaluation, although she denied any issues with tinnitus or adenopathy/adenitis, she endorsed a wide range of other, chronic/recurrent, episodic/intermittent and/or waxing/waning issues including subjective (rarely

objective) fevers, flushing, feeling cold much of the time, fatigue (often to the point of exhaustion), malaise, headaches, diffusely migratory aching/pain, diffusely migratory pruritus, unprovoked soaking sweats (mostly nocturnal), weight loss suspected to be due to two years of diarrhea and her dietary restrictions (but fairly constant hunger), irritation of the eyes, acute brief inability to focus vision (but ophthalmologic evaluations for this had all been negative), epistaxis (both nares had to be cauterized), easy bleeding, easy bruising, sinonasal congestion, coryza, post-nasal drip, intranasal sores, oral pruritus, occasional sore throats, fear of dysphagia (but not dysphagia itself), dyspnea (acute spells of this had driven her to the ED, where evaluations had consistently been unrevealing), palpitations, non-anginal chest discomfort/pain, gastroesophageal reflux in the past, nausea, rare vomiting, diarrhea alternating with constipation (much more so the former), diffusely migratory abdominal discomfort including (usually post-prandial) bloating, urinary frequency, diffusely migratory weakness, a sense of diffusely migratory edema in her joints but no physically visible edema, diffusely migratory tingling/numbness (typically about the distal extremities), orthostatic and non-orthostatic presyncope, syncope, cognitive dysfunction (particularly memory, concentration, and word-finding), waxing/waning hair loss (sometimes severe), odontalgia despite good attention to dental hygiene, poor nail growth, diffusely migratory rashes (typically patchy macular erythema), “hives all the time” (most commonly on her abdomen), insomnia, frequent waking, non-restorative sleep, hypersomnolence, sleeptalking, occasional sleep paralysis, panic disorder, anxiety, laxity in multiple joints (but a spontaneously demonstrated Beighton score of 0/9), unusually vigorous reactions to insect bites (especially mosquito bites), and poor healing in general.

Family history was notable for a mother with obesity, a hepatic hemangioma, and polycystic ovarian syndrome (PCOS), a father who had asthma, hypertension, arthritis, and bilateral hearing loss, a brother who had asthma and severe peanut allergy, a paternal grandmother who had had a penicillin allergy and died of complications of steroid-treated rheumatoid arthritis at 42, a paternal grandfather who had arthritis, asthma, and allergies to peanuts and chocolate and had had a stroke at 91, a maternal grandfather who had had hypertension and a dissecting aorta and then died from a pulmonary embolism five days after surgery for the dissection, a maternal grandmother who had obesity, asthma, alcoholism, and a sleep disorder. The patient denied any history of smoking, significant alcohol use, or illegal substance use.

At the time of the initial evaluation, the patient listed her current medications as sertraline 50 mg once daily and a daily oral contraceptive taken principally to address her PCOS. She listed her current allergies as hives to amoxicillin and "feeling horrendous" to prednisone and budesonide. Outside records indicated drug allergies to penicillins and sulfa.

Physical examination at the initial evaluation found an outwardly healthy appearing, trim young woman in no apparent distress, pleasant, cooperative, fully alert and oriented, easily independently ambulatory, presenting with her obviously appropriately concerned/supportive mother. Vitals were notable for BP 117/77, pulse 93, and weight 116 pounds (down from a peak of 140, she said). Key findings were her comfortable general appearance, HEENT benign, no plethora/pallor/diaphoresis/jaundice/bleeding/bruising, a very minimal extent of folliculitis about the forehead and upper back but otherwise no rashes at all (nor any hives), neck supple, no JVD or thyromegaly or carotid/abdominal/renal/femoral bruits, no palpable adenopathy or tenderness

at any of the usual node-bearing sites, clear lungs, regular heart with no adventitious sounds, abdomen scaphoid and completely benign, no peripheral edema, and an unremarkable neurological exam. No costovertebral angle or spinal column tenderness was evoked on palpation and percussion about these areas. On a light scratch test on the upper back, mildly bright dermatographism (erythroderma only, no hives) quickly emerged and was fully sustained when last checked 10 minutes later.

Case 3

This female, who was 24 at the time of her initial presentation for evaluation for MCAS, had frequent "colds" in infancy, and in elementary and middle school she suffered frequent "Strep infections," though with some of these incidents, she did not have very significant symptoms, even though the testing was positive for Strep anyway. Menarche came at 13, and she was immediately afflicted by dysmenorrhea and menorrhagia. At 15 she was started on doxycycline for acne, but this did not help much. At 17, in her senior year of high school, soon after being taken off doxycycline, she started getting frequent "stomachaches and cramping" and waxing/waning diarrhea alternating with constipation. Shortly after starting college, her constipation started worsening and she began suffering new troubles with allergies. She also started suffering episodes of idiopathic urticaria about her trunk and extremities. She was tried on antihistamines, which helped the hives but not the gastrointestinal (GI) issues. She also began suffering chronic fatigue. She consulted a naturopath, who advised she try an egg- and dairy-free diet, which she found did help her GI symptoms somewhat, albeit incompletely. She consulted an allergist about these other symptoms and her waxing/waning periorbital edema.

Her allergist performed skin patch allergy testing, which showed only a cobalt allergy and a variety of seasonal allergies. She then started getting subcutaneous immunotherapy at 19, which did help somewhat with her allergies, but then at 21, her episodes of urticaria worsened, accompanied by throat closing and numbness and pain about two hours after each immunotherapy treatment, so she stopped this treatment at that time. At 22, she got her first Gardasil vaccination. A little bit of facial edema ensued a few hours later and only lasted a few hours, but then she inexplicably lost 10 pounds over the next month. The second Gardasil vaccination was given on schedule a month after the first, and two days later she began feeling very dizzy and nauseous and started vomiting, and she soon came to be diagnosed with postural orthostatic tachycardia syndrome (POTS). She also noted that after the second vaccination, her chronic sinus congestion had worsened to include chronic bilateral ear congestion. Since the second Gardasil vaccination, too, she has been feeling a closing in her throat and the facial edema more often, and this tended to come on shortly after meals, especially with certain foods such as dairy, eggs, pineapple, and specific facial lotions, which also made her tongue feel as if she burned it. She had a similar problem emerge with certain medications and supplements such as a vitamin B12 supplement she had previously taken without difficulties, as well as with propranolol, quercetin, generic oral cromolyn, and pyridostigmine tablets (though she tolerated pyridostigmine syrup just fine). Pyridostigmine helped her POTS symptoms but not her other problems. Several months later, she consulted an allergist, who was the first to suspect she might have a mast cell activation disorder at the root of her troubles. However, the only testing performed was repeat skin patch testing, which only showed "a bunch of allergies" to dust, pollens, and trees. The allergist tried the patient on oral cromolyn, which again caused throat closing and tongue burning, so she was next tried on compounded oral ketotifen, which caused

sinus and ear congestion, itching, and then, a bit later, urinary frequency and urgency and pelvic pain which would briefly improve when she urinated. Repeated testing for a urinary tract infection was persistently negative. She then stopped the ketotifen, and the sinus and ear congestion, dysuria, and pelvic pain all resolved about a week later. At the time of her initial evaluation by author LBA, she reported chief complaints of food and medication/cosmetic reactivities, chronic fatigue, episodic acute dyspnea (explained as a sense that "I just can't catch a deep breath most of the time"), and sinonasal congestion. Additional past medical history identified in the medical records at the time of that initial evaluation included early satiety, occasional modest electrocardiographic abnormalities, acne, anxiety, mild intermittent asthma, dysfunctional uterine bleeding, bulimia nervosa, ovarian cysts, unspecified visual disturbances, fatigue, post-traumatic headache, herpes simplex labialis, urinary tract infection, abdominal wall pain, atypical chest pain, and pelvic pain. On a full review of systems at the initial evaluation, although she denied any issues with diaphoresis, easy bleeding, intranasal sores, wheezing, syncope, mental health problems (psychiatric evaluation found her problems were medical, not psychiatric), unusually vigorous reactions to insect bites, or healing problems, she endorsed a wide range of other, chronic/recurrent, episodic/intermittent and/or waxing/waning issues including subjective fevers, flushing, feeling cold much of the time, fatigue (often to the point of exhaustion), malaise, headaches, diffusely migratory aching/pain, diffusely migratory pruritus, unprovoked fluctuations in weight and appetite (she had been unable to get back to her normal weight since the Gardasil treatment, plus she reported early satiety), irritation of the eyes, acute brief inability to focus vision, tinnitus, epistaxis, easy bruising, sinonasal congestion, coryza, post-nasal drip, oral canker sores, burning tongue, irritation and numbness of the throat, proximal dysphagia, dyspnea, palpitations, non-anginal occasional chest discomfort/pain,

gastroesophageal reflux, nausea, vomiting, diarrhea alternating with constipation, diffusely migratory burning abdominal discomfort including (usually post-prandial) bloating, urinary frequency and urgency and dysuria as detailed above, diffusely migratory weakness (possibly secondary to pyridostigmine, she thought), periorbital and bilateral cheek edema, tingling/numbness (typically about her throat), diffusely migratory adenopathy and adenitis, orthostatic and non-orthostatic presyncope, cognitive dysfunction (particularly memory, concentration, and word-finding), hair loss since about age 17, deterioration of dentition despite good attention to dental hygiene, brittle nails, diffusely migratory rashes (typically patchy macular erythema), hives, insomnia (especially due to feeling hot and dyspneic and due to urinary urgency/frequency), frequent waking, hypersomnolence, and joint hypermobility.

Family history was notable for a paternal grandfather who had had a stroke and fatal lung cancer in his 80s (a few decades after he stopped smoking), a paternal grandmother who had glaucoma, a maternal grandmother who had Alzheimer's disease, heart problem and thyroid problems, macular degeneration, and a corneal detachment, a maternal grandfather who had macular degeneration and diabetes mellitus, a maternal aunt who had a thyroid problem, a paternal aunt who had autoimmune atrophic gastritis, a mother who had a benign thyroid growth with no impact on thyroid function, a father who had hypertension and atrial fibrillation, and a sister who had some allergies. The patient denied any history of smoking, significant alcohol use (beer caused a stomachache, wine caused flushing, and alcohol in general worsened her sinonasal congestion), or illegal substance use.

At the time of the initial evaluation, she listed her current medications as pyridostigmine syrup 60 mg thrice daily, levocetirizine 5 mg twice daily, olopatadine 0.6% nasal spray twice daily, and triamcinolone nasal spray 55 mcg twice daily. She listed her current allergies as throat closing to Augmentin, throat closing and tongue burning to propranolol and quercetin, throat closing and fever and presyncope and flushing and facial edema to adrenocorticotropic hormone (ACTH, Cosyntropin), and throat closing and tongue burning to generic pyridostigmine tablets. She sayid grated ginger or turmeric sometimes helped decrease her gastrointestinal problems. Outside records indicate throat swelling to amoxicillina/claviculanic acid and propranolol, faintness, dyspnea, and flushing to ACTH (Cosyntropin), upset stomach to dairy and eggs, and unspecified reactions to cobalt and neomycin on allergy testing.

Physical examination at the time of the initial evaluation revealed a trim young woman in no apparent distress, pleasant, cooperative, fully alert and oriented, easily independently ambulatory, presenting with her obviously appropriately concerned/supportive mother. Vitals were notable for a pulse of 87 and a temperature of 99.1F. Key findings were her comfortable general appearance, HEENT benign but for mild facial acne, no plethora/pallor/diaphoresis/jaundice/bleeding/bruising, a minimal extent of patchy macular erythematous rash waxing/waning/migrating about the neck, neck supple, no JVD or thyromegaly or carotid/abdominal/renal/femoral bruits except for a slight trace of an upper abdominal aortic bruit and a moderate left femoral bruit, no palpable adenopathy or tenderness at any of the usual node-bearing sites, clear lungs, regular heart with no adventitious sounds, abdomen scaphoid and completely benign but for mild epigastric tenderness to palpation, no peripheral edema, and an unremarkable neurological examination. No costovertebral angle or

spinal column tenderness was evoked on palpation and percussion about these areas. On a light scratch test on the upper back, moderately bright dermatographism (erythroderma only, no hives) quickly emerged and was almost fully sustained when last checked 10 minutes later.

Case 4

This female, who was 15 at the time of her initial presentation for evaluation for MCAS, was demonstrating eczema and allergies by age 3. Around age 10 she developed a barky dry cough which persisted, somewhat on and off, for about a year. Around age 12 she was diagnosed with asthma and started having frequent episodes of bronchitis. She was tested for allergies and started subcutaneous immunotherapy, which never seemed to help. In fact, she had significant adverse reactions to some of these treatments. Chronic fatigue began emerging, but testing for mononucleosis was negative. Soon she anaphylactically reacted to an immunotherapy treatment, and when this happened again with the next treatment two weeks later, these treatments were stopped. Several weeks later, while at a summer camp, she suffered a flu-like episode. A few months later, immunotherapy was tried again and again caused anaphylaxis. A few weeks later, at 14, she received her first Gardasil vaccination, "and that's when the floor just dropped out" (quoting from the patient) with marked worsening of fatigue (she often could not finish the schoolday), idiopathic anaphylactoid reactions every day, recurring fevers (hospitalization for one such episode led to extensive evaluation which was non-diagnostic), reacting to foods but testing negative for allergies to these foods, reacting to chemical odors/fragrances, chronic nausea, post-prandial reflux and vomiting, diarrhea alternating with constipation, hair falling out in clumps (diagnosed as alopecia areata), and frequent palpitations and severe presyncope (once

occasioning hospitalization for a heart rate of 180). Upper GI tract endoscopy was unrevealing, but she reacted to propofol and self-explanted her IV several times during the procedure. She was having frequent "bone pains" in her legs, once thought to be osteomyelitis, but this was disproven. At 15 she engaged a new pediatrician, who was the first to suggest that a mast cell activation syndrome might be the root issue. Diagnostic testing was strongly positive. Her diet had been reduced to only three tolerable foods, but she then consulted an immunologist and was started on oral cromolyn, famotidine, levocetirizine, and fexofenadine, whereupon she was able to significantly expand her diet. She soon consulted with an MCAS expert, who confirmed the diagnosis and recommended an increase in cromolyn and initiation of aspirin and omalizumab. She improved on aspirin, but omalizumab immediately worsened her fatigue and allergic reactions. Omalizumab was stopped after the second monthly treatment, and she improved. On a full review of systems at the initial evaluation by author LBA, although she denied any issues with tinnitus, easy bleeding, irritation of the mouth, dysphagia, chest discomfort/pain, genitourinary problems, paresthesias, adenopathy, syncope, onychodystrophy, mental health problems, joint hypermobility, or unusually vigorous reactions to insect bites, she endorsed a wide range of other, chronic/recurrent, episodic/intermittent and/or waxing/waning issues including subjective (rarely objective) fevers, flushing, feeling cold much of the time, fatigue (often to the point of exhaustion), malaise, headaches, diffusely migratory aching/pain, diffusely migratory pruritus, unprovoked soaking sweats (usually at night), unprovoked fluctuations in weight and appetite, irritation of the eyes, vision disturbances, epistaxis, easy bruising, sinonasal congestion, coryza, post-nasal drip, possible intranasal sores, frequent pharyngeal thrush and occasional throat sores, dyspnea/asthma (but had not needed nearly as much asthma medications since oral cromolyn began), palpitations, gastroesophageal reflux, nausea, vomiting, diarrhea

alternating with constipation, diffusely migratory abdominal discomfort including (usually post-prandial) bloating, diffusely migratory weakness, diffusely migratory edema (mostly about the face), diffusely migratory bilateral cervical adenitis, orthostatic and non-orthostatic presyncope, cognitive dysfunction (particularly memory, concentration, and word-finding), hair loss, unusual deterioration of dentition despite decent attention to dental hygiene, diffusely migratory rashes (typically patchy macular erythema), hives, eczema, hypersomnolence, sleep paralysis, and poor healing in general.

Family history was notable for healthy parents, a sister with allergies, another sister with high-histamine-content food sensitivities and vigorous reactions to bee stings, a paternal grandfather who had had fatal colon cancer in his 70s, a paternal grandmother who had had fatal heart failure, a maternal grandfather who had had fatal esophageal cancer at 85 (but who had not been a smoker or heavy drinker), a maternal grandmother who had had fatal ovarian cancer at 73, and a maternal aunt who had had breast cancer in her early 60s and after that developed “a lot of funny skin reactions.” The patient denied any history of smoking, significant alcohol use, or illegal substance use.

At the time of her initial evaluation by author LBA, she listed her current medications as cyproheptadine 4 mg qd, generic oral cromolyn 200 mg po qid, levocetirizine 5 mg qam, brand-name Allegra 180 mg bid, famotidine 20 mg bid, and aspirin 40 mg qd. She listed her current allergies as hives to doxycycline and Bactrim and other unspecified reactions to “a lot of antibiotics,” but she did not react to amoxicillin.

Physical examination at the time of her initial evaluation found a vaguely wan/fatigued-appearing, trim young woman in no acute distress other than intermittently exhibiting modest headache behaviors, pleasant, cooperative, fully alert and oriented, easily independently ambulatory, presenting with her obviously appropriately concerned/supportive mother. Vital signs were notable for a pulse of 103. Key findings were her vaguely constitutionally unwell general appearance, HEENT benign, no plethora/pallor/diaphoresis/jaundice/rash/bleeding/bruising, diffusely mild dryness all about her skin, neck supple, no JVD or thyromegaly or carotid bruits, no palpable adenopathy or tenderness at any of the usual node-bearing sites, clear lungs, regular heart with no adventitious sounds, abdomen completely benign, zero peripheral edema, neuro grossly intact. No costovertebral angle or spinal column tenderness was evoked on palpation and percussion about these areas. On a light scratch test on the upper back, mildly bright dermatographism (erythroderma only, no hives) quickly emerged and was fully sustained when last checked 10 minutes later.

Case 5

At her initial evaluation for MCAS, this 21-year-old female reported she had had a lifetime (literally from birth to present) of "always having a cold" and "always throwing up." She also was treated for what was thought to be a urinary tract infection at age 1 year. Early evaluations by her pediatricians led to diagnosis of gastroesophageal reflux disease (GERD). She also "passed out sometimes from crying too hard." She otherwise seemed healthy. At 6, she suffered a bout of pertussis in spite of having received the vaccine on schedule. Her mother noted the

patient "would always be late to elementary school because she'd be throwing up and having constipation sometimes, diarrhea other times." At 10, when she was only at the 5th percentile for weight, she started consulting with a pediatric gastroenterologist, who again diagnosed GERD. She was started on lansoprazole, which helped "a little," but she was never truly well. Celiac disease investigations were negative. Menarche came at 12, and she was immediately afflicted by dysmenorrhea and menorrhagia. At 13, upon having to stand for a long time, she suffered a full syncope. Other orthostatic hypotensive events ensued occasionally. Around age 15 she not only started an oral contraceptive, which did help control these problems, but she also was started on cyproheptadine as an appetite stimulant, and she found that helpful "for a number of years." Her mother noted, however, that "when she was great, she wasn't 'great,' but at least she wasn't dysfunctional." She even got to the point where she could skip her dosing of cyproheptadine for 1-2 days, but then nausea would relapse as a reminder that she needed to resume this drug. At 15 she got her first Gardasil vaccination, and she got the second one, too, on schedule. There were no early adverse reactions to either of these first two Gardasil vaccinations. She was delayed in getting the third. About three months after the second injection, she accidentally tripped, fell down a flight of stairs, and suffered a concussion. More GI symptoms emerged in the next week (more constipation, more abdominal pain, more vomiting) as well as urinary symptoms (marked frequency). At 16 she got her third Gardasil vaccination, and within the next month, episodes of orthostatic hypotension worsened. She was referred to a cardiologist, who made a clinical diagnosis (based on orthostatic maneuvers, but without formal tilt table testing) of POTS. She was also having presyncope (idiopathic and at micturition) and tachycardic palpitations. A brain MRI was negative. She initially refused offers of medication trials and just tried salt and electrolyte and water supplementation, which only

modestly helped.  She began identifying triggers of flares such as heat, prolonged standing, and smoke and other odors.  She changed to a gluten-free diet, which did not make a significant difference.  At 18, in her senior year of high school, when she was having “many ED visits and CT scans for abdominal pain” (none of which ever identified a problem except once when she was found to have a kidney stone, which was removed; she was also found to have a liver mass, thought to be benign (fibronodular hyperplasia), around this time), she underwent a “nutritional cleanse” involving “special supplements” and a “very limited diet” for 60 days, which stopped her nausea, vomiting, diarrhea, and constipation.  Shortly after completing this cleanse, she suffered a week-long bout of swelling about most of her major joints; orthopedic and rheumatologic evaluations were unrevealing.  Later at 18 she went away to college.  Literally while unpacking in her dormitory room at college, all of her symptoms relapsed.  She says that after one semester (during which she had “continuous nausea and vomiting” and “frequent ED visits”), she had to return home.  Soon after returning home, she suddenly found herself unable to swallow.  She underwent upper GI tract endoscopy, which reportedly found only modest candidal esophagitis.  She was treated with oral fluconazole, which seemed to help her dysphagia, but not her other symptoms.  She then attended a “program for POTS” which treated her with an unclear assortment of supplements and biofeedback.  She saw modest improvement in some of her symptoms.  A few months later, she was diagnosed with mononucleosis. Curiously, the initial few rounds of testing were negative, but then another round turned up positive.  However, she did not appear to have gross cervical adenopathy, let alone splenomegaly.  She then enrolled in another college as a commuter student.  At 19 she was diagnosed with anxiety and was tried on sertraline.  She could only take it for two nights, as it immediately caused a sense of severe pressure inside her head, even though no abnormalities

were seen externally. She re-tried the same supply of sertraline a few months later and again immediately experienced the same adverse effect and again had to quickly stop it. However, this re-trial of sertraline seemed to ignite a chronic sense of swelling and pain all throughout her lower oral tissues and neck. She reported this swelling was sufficiently severe to cause her to often bite her cheeks. However, multiple evaluations (dentist, oral surgeon, ENT, neurology, and immunology) all failed to identify any abnormalities. She also underwent extensive allergy testing and autoimmune testing, also all negative. As time went on, she went "downhill" with worsening nausea, vomiting, and sensitivities to various foods. She was unable to drive and had to be driven to school. In the month before initial evaluation by author LBA, she required hospitalized for dehydration because she could not swallow or eat and had lost 8 pounds in about one week. She was started on amitriptyline in the hospital and took it for a week, but it was caused a dry mouth and worsening her POTS symptoms (specifically, supine tachycardia worsened to 155), so she was switched to escitalopram and found this helpful for her tachycardia. She also was started on fludrocortisone for her POTS while in the hospital, but she quickly found this caused bad migraine headaches and resumption of menstruation (which normally was fully suppressed by her oral contraceptive) and severe muscle cramps so severe she could barely walk, so this drug trial was stopped after two weeks. Through her own research, the patient came to suspect that a mast cell activation disorder might be at the root of her illness, leading to her seeking evaluation by author LBA. At the time of that initial evaluation, she reported chief complaints of dyspnea, vomiting, tachycardia, and belching. Other past medical history noted at that time included beta thalassemia minor, biliary dyskinesia, gastroparesis, and chronic aerophagia. On a full review of systems at the initial evaluation, although she denied any issues with diaphoresis, vision anomalies, epistaxis, easy bleeding, easy bruising, intranasal

sores, wheezing, abdominal bloating, hair/nail problems, hives, joint hypermobility, and unusually vigorous reactions to insect bites, she endorsed a wide range of other, chronic/recurrent, episodic/intermittent and/or waxing/waning issues including subjective (rarely objective) fevers, flushing, feeling cold much of the time, fatigue (often to the point of exhaustion), malaise, headaches, diffusely migratory aching/pain, diffusely migratory pruritus, unprovoked fluctuations in weight and appetite, irritation of the eyes, tinnitus, sinonasal congestion, coryza, post-nasal drip "all the time," irritation of the mouth, irritation of the throat, proximal dysphagia, dyspnea ("I feel really tight at times in my throat and lungs, but I'm told my airway is wide open and my oxygen is at 100%"), palpitations, non-anginal chest discomfort/pain, gastroesophageal reflux, nausea, vomiting, diarrhea alternating with constipation, diffusely migratory abdominal discomfort, urinary frequency, occasional dysuria (she said it would always feel like she has a UTI, but evaluation for such was always negative; she said she had had only one true UTI in her adolescent/adult life), diffusely migratory weakness, subjective edema about the oral/cervical tissues as described above but never any objective edema (except for the week-long bout of joint swelling previously noted), diffusely migratory tingling/numbness (typically about the distal extremities), diffusely migratory bilateral cervical adenitis (but not adenopathy), orthostatic and non-orthostatic presyncope, syncope rarely (as detailed above), cognitive dysfunction (particularly memory, concentration, and word-finding), some aberrant dental growth requiring multiple oral surgeries, diffusely migratory rashes (typically patchy macular pruritic erythema), insomnia, frequent waking, hypersomnolence, non-restorative sleep, sleepwalking in childhood, sleeptalking, sleep paralysis, night terrors, anxiety, panic, depression, and poor healing in general (principally excessive scarring).

Family history was notable for a mother who had thalassemia minor, anxiety, depression, panic, and environmental allergies, a father who had a seizure disorder, hypertension, fluctuating kidney function, atrial fibrillation during his seizure, obstructive sleep apnea (wears CPAP), easy bruising from preventive aspirin, frequent kidney stones, and a penicillin allergy, a sister who had "borderline renal hypertension" but was otherwise healthy, a paternal grandfather who had had hypertension and had died of kidney failure, a paternal grandmother who had hypertension, diabetes, and hypertrophic cardiomyopathy and had had a breast cancer at 78, a maternal grandfather who had multiple myeloma and hypercholesterolemia and had undergone coronary artery bypass at 65, and a maternal grandmother who had hypercholesterolemia, hypertension, fatty liver disease, frontal lobe dementia since her 50s (and now had Alzheimer's disease), anxiety, depression, and had had uterine cancer in her 70s. The patient denied any history of smoking, significant alcohol use (in fact, it immediately caused her to experience nausea, vomiting, and lightheadedness), or illegal substance use.

At the time of her initial evaluation, she listed her current medications as pantoprazole 40 mg once daily, ondansetron 4 mg as needed for nausea, Ortho Micronor 0.35 mg once daily as an oral contraceptive, and escitalopram 5 mg once daily. She listed her current allergies as sertraline. Outside records indicated a "head in vise" feeling to sertraline, extrapyramidal symptoms to metoclopramide, and reactivities to nickel, gluten, and pepper.

Physical examination at the time of the initial evaluation found an outwardly healthy appearing, trim young woman in no apparent distress, pleasant, cooperative, fully alert and oriented, easily

independently ambulatory with no apparent orthostatic behaviors, presenting with her obviously appropriately concerned/supportive parents. Vital signs were unremarkable but for a weight of 101 pounds. Key findings were her comfortable general appearance, HEENT benign, no plethora/pallor/diaphoresis/jaundice/rash/bleeding/bruising, neck supple, no JVD or thyromegaly or carotid/abdominal/renal bruits (but she did have soft bilateral femoral bruits), no palpable adenopathy or tenderness at any of the usual node-bearing sites, clear lungs, regular heart with no adventitious sounds (but the degree of variability in her heart rate during the respiration cycle was a bit more pronounced than usual), abdomen completely benign, no peripheral edema, and an unremarkable neurological exam. No costovertebral angle or spinal column tenderness was evoked on palpation and percussion about these areas. On a light scratch test on the upper back, moderately bright dermatographism (erythroderma only, no hives) quickly emerged and was fully sustained when last checked 10 minutes later.

Laboratory testing for mast cell activation was positive for a 24-hour urinary prostaglandin $D_2$ level elevated at 432 ng (normal 100-280) and a plasma heparin level strongly elevated at 0.17 anti-Factor Xa units/ml (upper normal 0.02 [1]). Comprehensive genetic testing for autoinflammatory syndromes was negative.

Trials of various non-sedating $H_1$ blockers and various $H_2$ blockers found fexofenadine and ranitidine served her better than the others, having gained her a broad range of symptomatic improvements at follow-up three months after initial evaluation, including being much more energetic (engaging in more routine activities of daily living) and enjoying complete resolution of her previous daily emesis. She was able to complete college and is pursuing graduate

schooling. At the time of the broad ranitidine recall in the U.S. in 2020, she switched to famotidine with only modest loss of efficacy.

Case 6

At her initial evaluation for MCAS, the patient was a 23-year-old female who had a history of recurrent diffusely migratory joint pain since age 10 and who had experienced her first syncopal event at age 15, resulting in a foot fracture from the fall. Also in adolescence, she began suffering unexplained fatigue and occasional episodes of (mostly orthostatic) dizziness and tachycardia. She was in this ("usual") state of health until she received her first Gardasil injection at age 18, which resulted in flu-like symptoms, hives, and itching within the next day and lasted about a week. She received the second Gardasil injection six weeks later and, one day later, developed worsening of the initial post-Gardasil symptoms to the extent that she decided not to receive the third dose. She also began noticing some worsening of her prior fatigue and episodes of orthostatic dizziness and tachycardia, and the worsening of these symptoms steadily progressed over the next several years. Three months after the second injection, she developed new allergic reactions to various foods as well as gastrointestinal (GI) symptoms such as vomiting, abdominal pain, and daily nausea despite no apparent triggering exposures. At 19 she consulted a gastroenterologist for a new type of abdominal pain. She was started on omeprazole and esomeprazole for presumed gastroesophageal reflux disease (GERD), but these treatments did not improve any of her symptoms. She remained unable to digest vegetables and most other plant-based foods and was able to eat only meat, squash, and bananas, but she found this extremely limited diet helpful in terms of ameliorating some of her symptoms. Also at 19, the

joint pains she had suffered for nearly a decade finally led to evaluation by a rheumatologist, who found she had a hypermobile cervical spine and a Beighton score of 9/9. She was diagnosed with hypermobile Ehlers Danlos Syndrome (hEDS); physical therapy was recommended. At this point her medical problems required her to take a leave of absence from college, and she began working as a full-time receptionist, but with significant fatigue. At 22 she was evaluated by an allergist with skin testing, which was only borderline positive to only peanuts, dairy, coconuts, and egg yolk. For the orthostatic tachycardia, dizziness, and fatigue, she was referred for full autonomic function testing, which was positive for POTS and negative for small fiber neuropathy. Her chief complaints at the time of initial evaluation by SB were chronic joint pain, GI dysfunction, and recurrent hives, all exacerbated by diet. She also reported pruritus and headaches from smelling fragrances and a rash from swimming in pools. Her joint pains were primarily in her ribs and right shoulder; she also could easily dislocate multiple joints. She also had been suffering severe neck pain, which also had developed shortly after the Gardasil injections at age 18 and had been steadily worsening ever since. At initial evaluation by SB, she had lost 20 pounds over the prior 3-4 years due to her restrictive diet, which reduced her GI and allergic symptoms. She admitted to insufficient fluid intake on some days due to her GI symptoms. To try to address her orthostatic symptoms, she was consuming increased salt, which significantly helped. Trials of oral diphenhydramine 25 mg for her pruritus were not helpful. Also at the initial evaluation at 23, she reported frequent and painful urination, nocturia, and heat intolerance (including difficulty taking hot showers, with symptoms including lightheadedness and weakness). She reported a mild sleep deficit (6-7 hours per night) and also admitted to anxiety. She was taking sertraline and had tried other selective serotonin reuptake inhibitors (SSRIs) in the past, but none of them were helping her anxiety. She was started on low-dose

clonazepam to take on an as-needed basis, and this was somewhat helpful for her insomnia. She also noted her periods had been irregular since menarche. At the initial evaluation by SB, she reported she could go walking and shopping for limited extents; fatigue was her main limiting factor. She had not been able to return to college. Laboratory testing for MC activation was significant solely for an elevated serum prostaglandin $D_2$ at 211 pg/ml (normal 35-115). She also was found to have a positive anti-nuclear antibody (titer 1:320, homogeneous pattern), a beta-2-glycoprotein-1 IgG elevated five-fold above the upper limit of normal, an anti-cardiolipin IgG elevated three-fold above the upper limit of normal, and anti-thyroglobulin antibodies elevated 2.5-fold above the upper limit of normal. Anti-platelet therapy for possible anti-phospholipid antibody syndrome was considered, but she was allergic to non-steroidal anti-inflammatory drugs (NSAIDs), including aspirin. She managed her symptoms with a very restricted diet, avoidance of environmental triggers of flares of her symptoms, and high intake of fluids and salt. She was unable to find a tolerable antihistamine regimen because she was reactive to lactose and could not easily find or access lactose-free commercial or compounded formulations of these drugs. Oral cromolyn (200 mg four times daily), however, did significantly improve her oral mucosal tenderness and ulcerations and her flushing and pruritus, implying (given the poor absorption of this drug) that most of her dermatologic symptoms (and perhaps at least certain other symptoms, too) were the direct and/or indirect result of aberrant mediator expression by dysfunctional GI tract MCs.

Case 7

At the time of initial evaluation for MCAS, this 20-year-old male had been healthy and athletic, including for some time following receipt of his first Gardasil dosing at 16. However, two weeks after he received his second dose of Gardasil at 18, he began feeling sick in general and began experiencing unilateral knee redness and stiffness as well as a more generalized burning rash for the next three months. He managed to continue with work and school, though. Five months after the second Gardasil injection – at a check-up at which he complained about the knee pain and burning rash for the last few months – he received the third injection. Two days later, he heard his knees "pop" and felt his knees become weak and unable to bear his weight. He also developed burning pain in the legs, difficulty walking, and general fatigue. Efforts to "push" himself beyond his fatigue only made him feel even worse. By one month after the third injection, he required a cane to walk. At initial evaluation by SB, he described having "weak knees;" burning pain in his arms, face, and occasionally in the legs; numbness in his face, hands, and arms lasting from a few minutes to 1.5 hours; intermittent light-headedness and palpitations; recurrent headaches; difficulty concentrating and word-finding difficulty; and fatigue. He denied significant GI disturbance or urinary symptoms. He reported weight loss of 20 pounds over a period of a few weeks after he initially got sick, but he then was able to regain that weight. Due to his symptoms causing significant functional impairment, he had to take a leave of absence from college and also had to stop working. He had consulted many specialists, including a rheumatologist who diagnosed him with fibromyalgia. He was tried (at different points) on duloxetine and amitriptyline, which did not help. A tilt table test at age 20 was positive, and a diagnosis of postural orthostatic tachycardia syndrome (POTS) was made; he was instructed to increase salt and fluid intake, which he felt improved his light-headedness. At the time of initial evaluation by SB, his walking was limited to 30 minutes due to knee pain and fatigue. Bone

densitometry unexpectedly showed severe osteoporosis (T scores -4.0 and -4.1 in the bilateral hips). Evaluation by a geneticist for a question of Marfan syndrome was negative; whole exome sequencing was pursued, too, and was negative. Additional consultations were pursued with five endocrinologists, a rheumatologist, a metabolic bone disease specialist, and two orthopedic surgeons, all with no additional diagnostic insights. Due to persistent arthralgia causing difficulty walking and evidence of small fiber neuropathy (SFN) on quantitative sudomotor axon reflex testing (QSART), he was started on intravenous immune globulin (IVIG) at 2 g/kg every three weeks and experienced rapid, significant improvement in burning pain, weakness, joint pain, and ambulation; he was able to gain some weight, too. However, episodes of tachycardia, rashes, and severe osteoporosis all persisted, and the symptoms improved by IVIG reliably relapsed before his next IVIG dose was due. Laboratory testing for mast cell activation was significant solely for a plasma histamine level of 2.8 ng/ml (normal ≤ 1.8). Labs were also notable for mild decreases in serum copper (58 mcg/dl, normal 70-140) and iron (50 mcg/dl, normal 59-158) and low vitamin D (15 ng/ml, normal 30-100). His vitamin B12 level was low-normal (208 pg/ml, normal 200-910). Adrenergic and muscarinic antibodies (analyzed at Celltrend) were negative. Antihistamines gained him some improvements, but IVIG has persisted as his most effective intervention.

Case 8

At the time of initial evaluation for MCAS, this 19-year-old female had already had virtually a lifetime of multisystem polymorbidity. At age 3 she began showing behavioral rigidity and anxiety. She had three episodes of streptococcal pharyngitis as a child, treated with antibiotics.

Around age 8, she developed severe obsessive-compulsive disorder (OCD, involving symmetry and needing certain objects arranged in certain positions); this lasted six months and then spontaneously resolved. At 10 she was diagnosed with growth hormone deficiency and central precocious puberty. She had a histrelin acetate subcutaneous implant inserted at 10 and also was treated with growth hormone injections for three years. At 12 she developed bulimia, which was coincident with not only resumption of puberty but also suffering bullying at school. At 14 she became anorexic. Four months later, she started psychotherapy and consulted a nutritionist. Food intake soon normalized, but four months later she relapsed, requiring hospitalization for six weeks at age 15. Inpatient refeeding seemed to worsen her anxiety. She was tried on many anxiolytics, which did not help. Seven months after discharge, she relapsed and lost about 20 pounds of weight to 88 pounds, again requiring brief hospitalization followed by intensive outpatient follow-up, and then about three months later (at 16) she received the Gardasil vaccination series, each on schedule. Two months following the third injection, she developed migraine headaches, soon followed by irritable bowel syndrome with gastroparesis, abdominal pain, and constipation, which she never experienced previously. She began having difficulty tolerating food. Bidirectional GI tract endoscopy and colonoscopy was unremarkable. She was diagnosed with lactose intolerance and gluten intolerance, but despite changing her diet in accordance, her symptoms persisted. Digestive enzyme supplements were unhelpful. Testing for small intestinal bacterial overgrowth (SIBO) was negative. She soon developed severe depression. At 17 she was treated by an integrative psychiatrist for mood instability (especially in the pre-menstrual period) with a variety of pharmacologic and herbal anti-depressants without significant improvement. At 18 she went away to college, but three weeks later she developed severe dizziness and light-headedness, fatigue, and cognitive dysfunction ("brain fog"). Her

abdominal pain and constipation relapsed, too. The symptoms were so severe she had to leave school. She consulted with several physicians (including a neurologist, an otolaryngologist, and cardiologist (particularly for episodes of extreme tachycardia, with no specific findings on evaluation)) and was diagnosed, about three months after onset of these symptoms, with Lyme disease and briefly received treatment with oral antibiotics from the diagnosing physician before soon consulting at a large functional medicine center, where she was further treated with IV ceftriaxone for six months, seeing resolution of joint and muscle pain and fatigue. During this interval, she also was diagnosed by a dysautonomia specialist with POTS. A trial of fludrocortisone immediately proved intolerable. Propranolol was then tried but provided only minimal relief. Coincident with her POTS diagnosis, she also developed diffuse joint pain and weakness. Testing found positive fluorescent in-situ hybridization (FISH) for *Bartonella* species and a positive IgM Western Blot for relapsing fever *Borrelia*, so she was diagnosed with concurrent bartonellosis and borreliosis. (She had a history of multiple tick bites as a child but no history of the rashes classic for any tick-borne infectious diseases.) Initial treatment consisted of a rotating schedule of oral cefuroxime 500 mg twice daily, doxycycline 100mg twice daily, and azithromycin 500 mg once daily, but after three months, she had not shown any improvement. A percutaneously inserted central catheter (PICC) was placed and she then was treated for another six months with intravenous ceftriaxone 2 grams daily together with oral atovaquone 250 mg twice daily and proguanil 100 mg twice daily. With this regimen, her joint and muscle pains significantly improved, but her gastrointestinal symptoms, fatigue, dizziness, lightheadedness, and brain fog did not. She received psychiatric care for depression and anxiety. Genetic testing to investigate potentially relevant pharmacogenomic polymorphisms prior to treatment found heterozygous MTHFR-C677T, homozygous COMT-V168M, and an unspecified

heterozygous CACNA1C mutation as well. She was started on bupropion but did not tolerate it. She then was treated with nortriptyline 30 mg once daily, whose only benefit seemed to be slight improvement in her constipation. At initial evaluation by TTD at 19, she reported persistent fatigue, brain fog, dizziness, light-headedness, oligomenorrhea, and extreme mood swings. A two-week trial of an oral contraceptive worsened all her symptoms. She also complained of urge incontinence, dry mouth, heat intolerance, headaches, sensitivity to loud noises, irritability, depression and anxiety, lightheadedness, poor appetite, many food intolerances, burping, constipation, abdominal pain, and easy bruising. She was living at home with her parents and was not able to go to school or work at that time.

Her family history was remarkable for a mother with a history of infertility, premature menopause at age 43, and hypoglycemia. A younger sister had anxiety, depression, precocious puberty, growth hormone deficiency, and history of an eating disorder. Her maternal grandmother had thyroid nodules. Her paternal grandmother had osteoporosis.

Physical exam was notable for dark circles under her eyes (consistent with “allergic shiners”) and lentigo reticularis about her legs. Dermatographism was seen on skin exam. Orthostatic vital sign measurements revealed a symptomatic 20-point increase in heart rate from sitting to standing.

Initial laboratory evaluation by TTD was significant for repeatedly elevated plasma histamine and chromogranin A levels (histamine: 10 ng/ml, 12 ng/ml, 10 ng/ml, and 12 ng/ml checked over several months (normal 0-8); chromogranin A: 25 ng/ml and 26 ng/ml (normal ≤ 15, and without

any evident confounding factors including cardiac, renal, or hepatic failure, proximate proton pump inhibitor use, neuroendocrine malignancy, or chronic atrophic gastritis)). Serum tryptase levels were repeatedly stable in the low-normal range. She additionally was found to be positive for HLA-DQ2 but was never diagnosed with celiac disease. Repeated hemograms were notable only for persistent mild leukopenia (3200-3500/$mm^3$). Erythrocyte sedimentation rate was repeatedly mildly elevated (28-42 mm/hr, normal ≤ 20). A "Cunningham panel" (CellTrend) of testing for dysautonomia-associated autoantibodies found borderline to slight elevations in anti-CaMKinase, anti-dopamine receptor D1, and anti-tubulin titer.

Mast cell activation syndrome (MCAS), long pre-existing but likely exacerbated/escalated by Gardasil vaccination, was diagnosed. Initial efforts to find helpful histamine H1 and H2 receptor antagonists appeared unsuccessful. She could not tolerate loratadine, diphenhydramine, fexofenadine, famotidine, ranitidine, cetirizine, and levocetirizine, suffering worsening brain fog and "brain burning" with all formulations tried. Oral cromolyn was then tried and found well tolerated and slightly helpful for GI symptoms for two years but then had to be discontinued, as her OCD was worsening (in spite of no change in oral cromolyn formulation) and thought to be possibly attributable to cromolyn. Ketotifen was added, and she was able to tolerate up to 2 mg once daily with some improvement in food reactivity but then had to go down to 1.5 mg due to worsening OCD. Low dose naltrexone (LDN) was tried next (up to 3 mg once daily) but again worsened her OCD and require discontinuation. A generic formulation of imatinib was tried next at a low dose (100 mg once daily) but quickly worsened her brain fog and was soon stopped. Hydroxyzine and montelukast also caused severe brain fog and could not be tolerated. She then re-tried low-dose imatinib with the brand-name Gleevec formulation but again saw

quick worsening of brain fog as well as depression and had to stop it. Prucalopride was tried for her constipation, with only minimal improvement. BPC-157 injections were started and provided some relief for her constipation. She presently requires ongoing daily coffee enemas to evacuate. Meanwhile, around the time of her diagnosis of MCAS, neurologic consultation also led to a diagnosis of chronic inflammatory demyelinating polyneuropathy (CIDP). She was treated with IVIG 2 g/kg over two days per month. This treatment appeared to markedly improve her CIDP and initially improved her "brain burning" and "brain fog." However, over time she became more reactive to the treatment (with this reactivity appearing to be more lot-specific than brand-specific), and three months after the relapse of her OCD (not experienced since childhood), after about two years of IVIG treatment in total, this treatment was stopped. Three months later, a repeat Cunningham panel showed elevations in all markers except for the anti-dopamine receptor D2L titer. The anti-dopamine receptor D1 titer was 1:8000 (previously 1:2000, normal 1:500-1:2000), anti-tubulin 1:4000 (previously 1:2000, normal 1:250-1:1000), and anti-GM1 titer 1:320 (normal 1:80-1:320). CaM kinase II activity was one of the highest levels ever seen by the laboratory at 215% (normal 53-130%). These results were felt by the laboratory to be consistent with encephalopathy of possibly infectious or autoimmune etiology. Celecoxib 100 mg once daily, soon escalating to twice daily, was tried and was tolerated well but had no effect on her brain fog or OCD and was stopped. The only intervention which had an appreciable effect on her OCD was oral progesterone 100 mg once daily. This treatment immediately improved her OCD, but this improvement was lost after six weeks and was not regained even after increasing the dose to 200 mg once daily. Several courses of oral antibiotics were given after repeat testing for tick-borne infections showed elevated *Babesia duncanii* IgG antibodies and elevated *Bartonella henselae* IgG antibodies, but the patient had no response to

any of the regimens, including various combinations of rifampin, hydroxychloroquine, azithromycin, clarithromycin, sulfamethoxazole-trimethoprim, minocycline, and doxycycline.

As of March 2020 (age 22), her chief persistent symptoms include severe OCD, oligomenorrhea, and persistent constipation. The OCD has not responded to numerous psychopharmacological interventions, including vilazodone, lamotrigine, and lurasidone. Alprazolam has been only mildly helpful for her anxiety. She is completely disabled from OCD. She is being evaluated for ketamine infusions.

Case 9

At the time of initial evaluation for MCAS, this 27-year-old female reported having been noticed by her mother at birth to have somewhat enlarged genitalia. Dermatographism was apparent from early in infancy; she was very sensitive to certain clothing and tags. Her sleep cycle has been unusual her entire life, dating to infancy: she often slept best after 1:00 a.m. and slept later in the morning. At age 2, normally active and verbal, she developed a severe reaction to her fourth Tdap (tetanus, diphtheria, pertussis) vaccine, a global paresis (unable to walk) and aphasia which lasted several hours and spontaneously resolved. The reaction was attributed to the pertussis component of the vaccine, so the next vaccine she received in this series was DT without pertussis, and no reaction was seen. Early in childhood, she developed cold-induced asthma and an episode of hyperactivity after ingesting a red-dyed food. She began manifesting frequent/chronic ear infections and headaches as well as recurrent stomach pains and nausea, which were attributed to alleged, but never proven, lactase deficiency. At 11, menarche was

unremarkable. She was otherwise "healthy" until age 14, when she developed a "post-viral syndrome." Following a non-specific viral-like illness, she experienced severe fatigue and needed to nap for three hours after school. Photophobia and mydriasis emerged and have persisted ever since. Severe dysmenorrhea emerged, and an oral contraceptive was started for presumed endometriosis. Migraines, Raynaud's phenomenon, presyncopal spells (typically orthostatic), and intermittent urticaria to hot water also emerged. Fatigue eased somewhat over months (she felt she was "very functional" for the rest of high school) but was circadian, worse in the mornings. At 16 she had her first syncopal episode and could no longer stand in one position for any significant time without becoming pre-syncopal. At 18, in her first year at college, she developed episodes of stabbing leg pains, paresthesias in her extremities, and chest pain at the superior aspect of the rib cage, all of which have persisted ever since, though frequency diminished later with treatment for a diagnosis of Lyme disease (no tick bite or bull's-eye rash was ever seen). Also in her first semester at college, she developed frequent aphthous oral ulcers and scalp folliculitis. She then "fell apart" during her second semester. She developed severe brain fog and fatigue, could no longer keep up with her schoolwork, and had to return home. She was soon diagnosed with mononucleosis and then Lyme disease. She took a short course of doxycycline but had difficulties tolerating it. She completed her schoolwork that semester from home with difficulty. Still symptomatic, she returned for her sophomore year, and in the spring semester, at age 19, she received the series of three Gardasil vaccinations. One month after her last Gardasil vaccination, she received the Yellow Fever vaccine to prepare for a school-related trip to Argentina but only had some local erythema to this vaccination. She traveled to Argentina two months later (i.e., three months following her last Gardasil vaccination) and soon developed severe gastritis. She then soon developed a severe ear infection

with rupture of the tympanic membrane and was treated with an antibiotic. Soon after the ear infection, she developed more brain fog and fatigue, albeit with different senses of these symptoms than previously experienced. Post-prandial tachycardia episodes and worsenings of fatigue emerged. She became "more sensitive" to her environment in general, including odors, sounds, and lights. Headaches worsened. She increasingly needed social isolation. At 20 she was re-treated for Lyme disease with high-dose amoxicillin, with little apparent benefit. At 21, in her senior year in college, she developed severe motion sickness, which has persisted ever since. Severe chemical sensitivities emerged and have persisted ever since. At 24, she noticed she had become completely intolerant of alcohol. Because of increasing gastrointestinal symptoms as well as a new diagnosis in her younger sister of non-celiac gluten intolerance, she decided to try a gluten-free diet, and though she lost weight, frequent nausea and bilious vomiting emerged, sometimes unrelated to food intake. She also developed aquagenic urticaria and cystic acne, which have persisted, waxing and waning, ever since. At 27, she suffered a pneumonia, requiring a course of azithromycin. Soon after, on initial evaluation by TTD, she reported severe fatigue, weight loss with cyclical vomiting, diarrhea with mucus, migratory joint pain, worsening facial and scalp cystic acne, trouble with concentration and memory, insomnia, increased urinary frequency, and mild alopecia. She also noted tachycardia and worsening concentration after meals that was so disruptive that she had to avoid eating before important assignments/tasks. Physical examination was unremarkable. Family history was notable for a younger sister who had polycystic ovarian syndrome (PCOS), a long history of chronic idiopathic urticaria, and presumptive mast cell activation syndrome (MCAS). In addition, her mother had recently been diagnosed with MCAS. Laboratory evaluation was notable for a low-normal vitamin B12 level, mild folate deficiency, mild vitamin D deficiency, a mildly elevated

high-sensitivity C-reactive protein (hsCRP), elevated HHV-6 IgG at 1:1280 but negative HHV-6 viral load by polymerase chain reaction (PCR), normal amylase and lipase, normal tryptase, a mildly elevated chromogranin A level (103 ng/ml, normal 0-95), a mildly elevated plasma histamine level (2.0 ng/ml, normal < 1.8), and a normal 2-4hour urinary N-methylhistamine level. Hormone testing, including evaluation for congenital adrenal hyperplasia, was negative.

MCAS-targeted therapy was initiated with trials of various non-sedating $H_1$ blockers. Loratadine 10 mg twice daily served her best but only provided some help for intermittent congestion. Ranitidine and famotidine both seemed to cause alopecia; she did not try any other $H_2$ blockers. Oral cromolyn 200 mg twice daily improved her nausea and vomiting, and her weight has stabilized. Medical marijuana, taken occasionally, also helps with symptom flares. She started low-dose naltrexone (LDN) and found 4.5 mg daily to be her optimal dose. Her hsCRP normalized. She has persistent fatigue and insomnia, worse in the spring. She is continuing with other MCAS-targeted medication trials.

Case 10

At the time of initial presentation for evaluation for MCAS, this 21-year-old female reported suffering, from early in infancy, significant constipation, frequent ear infections, dermatographism, and unusual sensitivities/reactivities to various sensations, including food textures, leading to a very limited diet. At age 5, she reacted to a water-color temporary tattoo. At age 7, although no tick bite was witnessed, a bull's-eye rash was seen on her. She was diagnosed with Lyme disease and treated with amoxicillin for two weeks. At age 8 misophonia

emerged. At age 12 aquagenic urticaria emerged. At 13 axillary folliculitis developed from shaving. Menarche came at age 13; she has never had regular cycles. Focal vaginal pain precluded use of tampons. At 15 she received the full series of three Gardasil vaccinations. Three months after the last vaccination, she developed a non-specific illness, attributed to an unidentified viral infection. Within days exhaustion emerged and has persisted ever since. Motion sickness emerged, too, and continued for several years before spontaneously resolving. Dysmenorrhea emerged, and she was clinically diagnosed with endometriosis. Depression emerged. A trial of duloxetine did not help and only worsened her fatigue. Methylphenidate was tried but only worsened her misophonia. She could not concentrate in class from being too disturbed by merely the breathing of those around her. At 16, about a year after completing the Gardasil series, she was diagnosed with Lyme disease again. A prolonged course of doxycycline seemed to ease her fatigue somewhat, but she also began experiencing eczema and a burning sensation about her skin. Topical hydrocortisone helped somewhat. At 17, her dietary constraints seemed to ease somewhat. At 18 she was diagnosed with scoliosis; a course of physical therapy was undertaken, to no clear benefit. She also was started on an oral contraceptive for her irregular periods but soon developed severe irritability and depression. Evaluation discovered elevated cortisol. Multiple endocrinologic evaluations, including contrasted brain/pituitary MRI, failed to identify a cause. She developed severe hives and dizziness from the MRI contrast. The oral contraceptive was discontinued, and the irritability, depression, and cortisol elevation resolved. Further evaluation for weight gain, irregular menses, acne, and mild male-pattern alopecia (without hirsutism) led to a diagnosis of polycystic ovarian syndrome (PCOS). She was treated with spironolactone and intermittent progesterone to induce menses. She was also diagnosed with hypothyroidism and was treated with a compounded

T3/T4 preparation.  At 19 bouts of vertigo and post-prandial tachycardia emerged, fatigue worsened, and nausea and anorexia emerged.  Laboratory testing confirmed non-celiac gluten sensitivity.  She eliminated gluten from her diet, which helped some of her gastrointestinal symptoms, but overall she continued to be quite symptomatic.  At 21 she started having allergic reactions, with erythema and pain, to application of deodorant.  She determined the baking soda in the product was the trigger.  She was initially evaluated by TTD at 21 for complaints of persistent fatigue, difficulty concentrating, depression, amenorrhea which had persisted for six months, and frequent urticaria from multiple triggers.  She was being treated by another physician at that time for multiple concurrent infections (Lyme disease, ehrlichiosis, bartonellosis, and babesiosis) with several antibiotics including dapsone, azithromycin and rifampin, to no clear benefit.  Laboratory testing was notable for a normal total IgE level, an off-the-scale elevated anti-IgE-receptor antibody level, a normal anti-IgE antibody level, normal tryptase, normal chromogranin A, mildly elevated plasma histamine (2.2 ng/ml, normal 0.0-2.0), and a normal diamine oxidase level.  MCAS-targeted treatment was initiated with trials of various $H_1$ blockers.  She had a moderate response to loratadine.  Various $H_2$ blockers were tried but were all proven intolerable due to worsening of alopecia.  She was next treated with low dose naltrexone (LDN), with some improvement in her depression.  Oral cromolyn significantly improved her nausea and intermittent bloating.  Due to persistent urticaria, omalizumab was tried, and within three months she had a notable improvement in her fatigue and resolution of her urticaria, though these symptoms would noticeably relapse if treatment was delayed.  She has been stable on her regimen of loratadine, cromolyn, LDN, and omalizumab for two years, though increased fatigue is still noted at times of stress.

Case 11

At initial presentation for evaluation for MCAS, this 30-year-old female reported a decade or more of many slowly but steadily worsening complaints including abdominal pain, frequent nausea with vomiting, difficulty eliminating fecal matter from the rectum, episodic diarrhea with grease droplets and foul gas, great weight loss due to a severely limited diet, tinnitus, orthostatic tachycardia, fainting, flushing, itching, deep and migratory bone pain, and frequent anaphylaxis to many foods, medication products, and environmental exposures. She also reported frequent infections, constipation, asthma, migraines, and food sensitivities dating to her earliest memories. She developed idiopathic hirsutism at age 5 and was fully developed at age 10 with respect to height and female organs. She had hypermobile joints and came to be diagnosed with hypermobile Ehlers Danlos Syndrome (hEDS). At age 18, soon after coitarche and soon after initially testing positive or HPV, she was administered her first Gardasil 9 vaccine, developing hives and swelling near the injection site within hours. Orthostatic tachycardia emerged within four weeks after the second dose and became chronic, but it was a few years before she was formally diagnosed with postural orthostatic tachycardia syndrome (POTS), with pulse rate rising to 180 beats per minute upon standing. In her early 20's her constipation and body pain were diagnosed as pelvic floor dysfunction, which did not respond to physical therapy or a pain management program. At age 20 she presented with acute lower pain and hematuria. Computerized tomography with intravenous contrast caused nausea, vomiting, abdominal pain, pruritus, and rash. She newly noted the intravenous (IV) catheter was irritating (upon removal, a mottled rash was seen at the insertion site, but not where tape had been placed), and this continued to be the case with all subsequent IV catheters. An obstructing calcium oxalate

ureteral stone was extracted, and a plastic stent was placed. Pain and nausea from the stent were severe but resolved immediately upon stent removal. At ages 18 and 22, exploratory laparoscopies for ongoing abdominopelvic pain were done to look for endometriosis but failed to find such. During an ovarian cyst removal at age 29, endometriosis was incidentally found, involving 17 different areas including deposits near the ureters. During excision of these deposits, hydrodistension with cystoscopy showed interstitial cystitis with Hunner's lesions. A subcutaneous analgesic medication pump was inserted near the incision, but in the recovery room the patient noted severe pain at the pump site as well as nausea; these symptoms resolved as soon as the pump was explanted. Also in her 20s, she developed psoriasis and asthma. Throughout her 20s, she had severe attacks of upper abdominal pain. Evaluations included largely normal esophagogastroduodenoscopy (a duodenal biopsy was normal, though a gastric biopsy showed mild chronic inflammation without *Helicobacter pylori*), normal gallbladder ultrasound, and positive cholecystokinin-stimulated hepatobiliary iminodiacetic acid scan (0% ejection fraction). Gallstones were discovered at cholecystectomy. Plastic clips were used during surgery owing to her metal allergy. After surgery, upper abdominal pain continued. Endoscopic retrograde cholangiopancreatography demonstrated papillary stenosis, a normal bile duct, and nonspecific changes of the pancreatic duct. Sphincterotomy did not reduce the pain. Endoscopic ultrasound showed nonspecific pancreatic duct abnormalities but no definitive signs of chronic pancreatitis. Subsequent evaluation for lower abdominal pain included macroscopically and microscopically normal colonoscopy. Computerized tomography showed only some small non-obstructing right renal stones and a few loops of fluid-filled nondilated small bowel. At age 29 cervical adenocarcinoma in situ was diagnosed and treated with cone biopsy and loop electrosurgical excision procedure; vaginal swab was positive for high-risk

human papilloma virus (HPV) by polymerase chain reaction (PCR), but not serotypes 16 or 18/45. At age 30 she reacted to a trial of adalimumab for newly diagnosed psoriasis. Her diet had become limited to just chicken, two vegetables, and bread, as all other foods caused diffuse abdominal pain and nausea. Her BMI decreased from 17.5 in 2011 to 14.2 in 2021. She was also beginning to notice hair thinning (despite the earlier hirsutism) and vision issues (intermittent diplopia and depth perception problems). She was also beginning to notice hair thinning (despite the earlier hirsutism) and vision issues (intermittent diplopia and depth perception problems). The family history was remarkable for a brother with severe seasonal allergies, a mother with irritable bowel syndrome, seasonal allergies, recurrent eye irritation, and easy bruising, and a father with rheumatoid arthritis, flushing, and oxalate nephrolithiasis. Physical examination at presentation was remarkable for a chronically ill-appearing, underweight (BMI 14.4 kg/m$^2$) woman with a blood pressure of 179/90, a pulse of 60 which did not increase with standing, flushing about her chest, moderate diffuse abdominal tenderness, and hypermobile joints (Beighton score 8/9). MCAS was diagnosed at 32 upon recognition of her MCAS-consistent history and finding of an elevated 24-hour urinary *N*-methylhistamine level of 296 mcg/g Cr (normal 30-200) and an elevated serum chromogranin A of 146 ng/ml (normal < 102, and without confounding factors). Immunoglobulin G was quantitated at 691 mg/dl (normal 767-1590), and IgG subclass 1 was quantitated at 253 mg/dl (normal 341-894); other immunoglobulin classes and subclasses were normal. At age 32, MC-directed treatment was initiated. With cetirizine and famotidine twice daily, nausea, flushing, pruritus, sneezing, coughing, and rashes significantly decreased. Hydroxyurea [2] helped bone pain, and gabapentin helped neuropathic pain, tingling, abdominal pain, and sleep. Low-dose naltrexone (LDN) daily [3] helped joint pain, bone pain, joint swelling. Nortriptyline daily helped abdominal pain,

bilious diarrhea, and sleep. Salt supplements, midodrine, and propranolol helped her POTS (maintaining blood pressure and heart rate and reducing dizziness and fainting and nausea). Alprazolam came to be used as needed (helping with severe nausea and pain). Ondansetron came to be used as needed for nausea. Diphenhydramine and epinephrine were used as needed for flares and anaphylaxis. A pyridostigmine formulation was tried but caused severe abdominal pain; the drug was not re-tried in alternative formulations. An oral cromolyn formulation was tried but seemed to immediately cause severe global paresis; a compounded oral cromolyn formulation was also tried and again immediately found intolerable. Diet remains unimproved; she has proven intolerant of many nutrition supplementation products. No clinical signs of micronutrient deficiencies have emerged yet. Insurance approval for immunoglobulin supplementation (subcutaneous instead of IV due to catheter intolerance) is being sought.

References

1. Seidel, H.; Molderings, G. J.; Oldenburg, J.; Meis, K.; Kolck, U. W.; Homann, J.; Hertfelder, H. J. Bleeding diathesis in patients with mast cell activation disease. *Thromb Haemost* **2011**, *106*, 987-989, doi:10.1160/TH11-05-0351.

2. Afrin, L. B. Utility of hydroxyurea in mast cell activation syndrome. *Exp Hematol Oncol* **2013**, *2*, 28:1-28:8, doi:10.1186/2162-3619-2-28.

3. Weinstock, L. B.; Brook, J. B.; Myers, T. L.; Goodman, B. Successful treatment of postural orthostatic tachycardia and mast cell activation syndromes using naltrexone, immunoglobulin and antibiotic treatment. *BMJ Case Rep* **2018**, *2018*, bcr2017221405:1- bcr2017221405:6, doi:10.1136/bcr-2017-221405.
